# Suboptimal blood pressure control in chronic kidney disease stage 3: baseline data from a cohort study in primary care

**DOI:** 10.1186/1471-2296-14-88

**Published:** 2013-06-24

**Authors:** Simon DS Fraser, Paul J Roderick, Natasha J Mcintyre, Scott Harris, Christopher W Mcintyre, Richard J Fluck, Maarten W Taal

**Affiliations:** 1 Academic Unit of Primary Care and Population Sciences, Faculty of Medicine, University of Southampton, South Academic Block, Southampton General Hospital, Tremona Road, Southampton, Hampshire SO16 6YD, UK; 2 The Department of Renal Medicine, Royal Derby Hospital NHS Foundation Trust, Derby, Derbyshire, UK; 3 School of Graduate Entry Health and Medicine, University of Nottingham, Nottingham, UK

**Keywords:** Chronic kidney disease, Hypertension, Blood pressure control, Albuminuria, Diabetes, Primary care

## Abstract

**Background:**

Poorly controlled hypertension is independently associated with mortality, cardiovascular risk and disease progression in chronic kidney disease (CKD). In the UK, CKD stage 3 is principally managed in primary care, including blood pressure (BP) management. Controlling BP is key to improving outcomes in CKD. This study aimed to investigate associations of BP control in people with CKD stage 3.

**Methods:**

1,741 patients with CKD 3 recruited from 32 general practices for the Renal Risk in Derby Study underwent medical history, clinical assessment and biochemistry testing. BP control was assessed by three standards: National Institute for Health and Clinical Excellence (NICE), National Kidney Foundation Kidney Disease Outcome Quality Initiative (KDOQI) and Kidney Disease: Improving Global Outcomes (KDIGO) guidelines. Descriptive statistics were used to compare characteristics of people achieving and not achieving BP control. Univariate and multivariate logistic regression was used to identify factors associated with BP control.

**Results:**

The prevalence of hypertension was 88%. Among people with hypertension, 829/1426 (58.1%) achieved NICE BP targets, 512/1426 (35.9%) KDOQI targets and 859/1426 (60.2%) KDIGO targets. Smaller proportions of people with diabetes and/or albuminuria achieved hypertension targets. 615/1426 (43.1%) were only taking one antihypertensive agent. On multivariable analysis, BP control (NICE and KDIGO) was negatively associated with age (NICE odds ratio (OR) 0.27; 95% confidence interval (95% CI) 0.17-0.43) 70–79 compared to <60), diabetes (OR 0.32; 95% CI 0.25-0.43)), and albuminuria (OR 0.56; 95% CI 0.42-0.74)). For the KDOQI target, there was also association with males (OR 0.76; 95% CI 0.60-0.96)) but not diabetes (target not diabetes specific). Older people were less likely to achieve systolic targets (NICE target OR 0.17 (95% CI 0.09,0.32) p < 0.001) and more likely to achieve diastolic targets (OR 2.35 (95% CI 1.11,4.96) p < 0.001) for people >80 compared to < 60).

**Conclusions:**

Suboptimal BP control was common in CKD patients with hypertension in this study, particularly those at highest risk of adverse outcomes due to diabetes and or albuminuria. This study suggests there is scope for improving BP control in people with CKD by using more antihypertensive agents in combination while considering issues of adherence and potential side effects.

## Background

People with chronic kidney disease (CKD) are at increased risk of mortality, cardiovascular disease (CVD) and less commonly progression to end stage kidney disease (ESKD) [1,2]. Uncontrolled hypertension, albuminuria, and diabetes are independent risk factors for these adverse outcomes [3-8]. Hypertension is common in CKD, with estimates of prevalence between 60% and 92% in stage 3 [9-12]. Control of hypertension is arguably the most important intervention for reducing the increased risk of cardiovascular disease in people with CKD, and to slow progression to later stages of CKD [1,11-14]. However, there is evidence that optimum levels of blood pressure (BP) control are often not achieved among people with CKD, with consistent achievement of BP less than 140/90 observed in between 15 and 30% of patients (with as few as 13% achieving a 130/80 threshold) [15-17].

In the UK (as in many countries) early stages of CKD are principally managed in primary care. Several national and international guidelines recommend targets for optimal BP control in people with CKD but there are differences between them, including variation of the targets for those at higher risk of outcome (such as people with diabetes and albuminuria). In the UK there are National Institute for Health and Clinical Excellence (NICE) guidelines on the monitoring and management of CKD, and, in England, incentivised disease management targets from the primary care Quality and Outcomes Framework (QOF) [18,19]. NICE CKD guidelines set a BP control at target <140/90 mm Hg for most people with CKD or <130/80 in people with diabetes or high levels of albuminuria (ACR > 70 mg/mmol), while the QOF CKD BP target is ≤140/85 [19,20]. In the US, the National Kidney Foundation Kidney Disease Outcome Quality Initiative (NKF KDOQI) guidelines set a BP control target at <130/80 for all people with CKD [21]. The 2012 Kidney Disease: Improving Global Outcomes (KDIGO) guidelines for the management of blood pressure in CKD recommend that both diabetic and non-diabetic people with non-dialysis dependeant CKD with hypertension but without albuminuria should have BP controlled ≤140/90, and people with significant albuminuria (microalbuminuria or macroalbuminuria) with or without diabetes should control BP ≤130/80 [22].

Little is known about CKD-related hypertension control in primary care, particularly in individuals at higher risk, such as those with and without diabetes or albuminuria. In England QOF data are aggregated at practice level and do not allow for interpretation at individual level [23]. This study aimed to evaluate the factors associated with blood pressure control in a population of people with CKD stage 3 in primary care in the UK.

## Methods

### Participants and recruitment

Participants were recruited as part of the Renal Risk in Derby (RRID) study, a prospective cohort study of people with CKD stage 3 in a primary care setting. The methods for the RRID study have been published in detail elsewhere [24]. In summary, eligible participants were 18 years or over, met the Kidney Disease Outcomes Quality Initiative criteria for CKD stage 3 (estimated GFR [eGFR] of between 30 to 59 ml/min per 1.73 m^2^ on two or more occasions at least 3 months apart prior to recruitment), were able to give informed consent, and were able to attend their general practitioner (GP) surgery for assessments. People who had previously had a solid organ transplant or who were terminally ill (expected survival <1 years) were excluded. The RRID study is conducted by a single nephrology department, but participants were recruited directly from 32 GP surgeries. Eligible patients were invited to participate via a letter sent by their GP and telephoned the coordinating centre to schedule a study visit. Study visits were conducted at participating GP surgeries by the researchers.

### Data collection

First study visits were conducted from August 2008 to March 2010. Screening and baseline visits were combined due to the large proportion of elderly participants and the logistical challenges associated with conducting study visits in multiple primary care centres. Participants were sent a medical and dietary questionnaire as well as three urine specimen bottles, and were asked not to eat cooked meat for at least 12 hours before the assessment. Urine was collected as three early morning samples. Socioeconomic status (SES) was defined by two methods. First, using the Indices of Multiple Deprivation score (IMD); a social deprivation score comprising a composite measure of seven domains which demonstrates a strong relationship to health in all geographical locations [25]. Second, self-reported education status was collected; an important indicator of socioeconomic status in elderly populations [26]. Education status was categorised into eight groups (no formal qualifications, General certificate of Secondary Education (GCSE) or equivalent, Advanced level (A level), National Vocational Qualification (NVQ) 1–3, NVQ 4–5, first degree, higher degree, patient refused to answer), subsequently grouped into three for the purposes of analysis (group one: no formal qualifications, group two: GCSE or equivalent, A level, or NVQ 1–3, group three: first or higher degree, NVQ 4–5). Self-reported ethnicity information was collected, but due to the small number of non-white participants in this study, it was categorized into ‘White’ and ‘Other’ for the purposes of analysis.

At the assessment, information on questionnaires was checked, anthropomorphic measurements taken, and urinalysis performed. Blood specimens were taken and the three urine specimens were submitted for biochemical analysis. eGFR was calculated using the modified 4-variable Modified Diet in Renal Disease equation and categorised into four groups (>60, 45–59, 30–44, < 30) [27]. Albuminuria was defined as albumin/creatinine ratio (ACR) ≥2.5 mg/mmol in men ≥3.5 mg/mmol in women in at least two of the three urine specimens (≥3 mg/mmol in all people for the KDIGO guideline analyses). BMI was calculated from weight in kg divided by height squared in metres and categorised according to World Health Organization (WHO) categories underweight (<18.5 kg/m^2^), normal (18.5 – <25 kg/m^2^), overweight (25- < 30 kg/m^2^), and obese (> = 30 kg/m^2^) [28]. Central obesity was defined as a waist to hip ratio of ≥0.9 for men or ≥0.8 for women [29]. Diabetes was defined by having a previous clinical diagnosis in line with WHO criteria [30]. Previous cardiovascular event was defined as subject-reported myocardial infarction, stroke, transient ischemic attack, revascularization, or amputation due to peripheral vascular disease, or aortic aneurysm. Smoking status was categorized as never smoked, ex-smoker, and current smoker. Self-reported alcohol consumption was categorized by units per week as never drinking alcohol, drinking within recommended limits (<21 units for women, <28 units for men), and drinking above recommended limits. Blood pressure was measured after a minimum of five minutes rest in the sitting position, using a validated oscillometric device, recommended by the British Hypertension Society (Digital Blood Pressure Monitor Model UA-767, A&D Instruments Ltd, Abingdon, UK). The same device was used for all readings. BP was calculated as the mean of three readings that differed by <10%. Mean arterial pressure (MAP) was calculated as 1/3 the average SBP plus 2/3 the average DBP.

For the purposes of analysis, hypertension was defined as current antihypertensive medication, but those with a systolic BP >140 mmHg or diastolic BP >90 mmHg at baseline who were not on medication were also identified for descriptive purposes [31]. Target BP threshold was defined according to three clinical guidelines: the UK NICE guidelines BP target (<140/90 or <130/80 in people with diabetes and people with ACR > 70 mg/mmol), the US NKF KDOQI guidelines BP target (<130/80 for all people with CKD), and the KDIGO guidelines (≤140/90 or 130/80 in people with albuminuria) [19,21,22].

Participants were asked ‘Were you told that you may have an issue with your kidneys before you were contacted to take part in this study?’ Those answering ‘yes’ were defined as being aware of their CKD diagnosis. The study was approved by the Nottingham Research Ethics Committee 1. All participants provided written informed consent. The study was included on the National Institute for Health Research (NIHR) Clinical Research Portfolio (NIHR Study ID:6632) and was independently audited by QED Clinical Services in November 2009.

### Statistical analyses

In the population of people with hypertension on antihypertensive medication, standard descriptive statistics were used to compare the characteristics of people achieving and not achieving BP control by NICE, KDOQI, and KDIGO BP targets. Univariate and multivariable logistic regression (adjusting for age, sex, albuminuria, diabetes, CVD, and eGFR) was used to identify the factors associated with achievement of the three BP targets. A model excluding CVD was also constructed to assess the effect of this variable on outcomes in view of the potential for CVD to cause lower BP through heart failure. Sensitivity analyses were conducted in participants whose baseline eGFR was <60 ml/min/1.73 m^2^. The logistic regression analyses were also repeated to examine the associations of people achieving NICE systolic and diastolic targets separately. Interaction terms were introduced for gender by diabetes, age by diabetes, and diabetes by albuminuria because of the effect modification seen among these variables in some studies [32]. Chi squared test for trend was used to examine the degree of BP control by grade of albuminuria in people with and without diabetes. For people on antihypertensive medication or those with elevated BP identified at study registration, multivariable linear regression was used to investigate the association between number of antihypertensive medications and MAP. All odds ratios are presented with 95% confidence intervals (CIs) and p values <0.05 are considered statistically significant. IBM SPSS Statistics for Windows version 19 was used to analyse the data.

## Results

### Study population

22% (1741) of approximately 8280 eligible participants from 32 GP practices invited to be included in the study agreed to participate (range 8-34% in different GP practices) and attended baseline assessment. Mean eGFR was 52.5 mL/min/1.73 m^2^ (SD 10.4). 418 (24%) people had eGFR ≥ 60 at baseline assessment. 280 (16.1%) had albuminuria on two of three ACR measures. 1426 (81.9%) were taking antihypertensive medication and a further 102 (5.9%) had high BP at study assessment (Table 1). See Additional file 1: Table S1 for characteristics of people with and without hypertension.

**Table 1:** Characteristics of people in the RRID study

	**Category**	**Total n = 1741 (numbers are n (% of total) unless otherwise stated)**
**Gender**	Male	689 (39.6%)
	Female	1052 (60.4%)
**Age**	<60	128 (7.4%)
	60-69	445 (25.6%)
	70-79	761 (43.7%)
	80+	407 (23.4%)
**Ethnicity**	White	1698 (97.5%)
	Other	43 (2.5%)
**Index of multiple deprivation**	Quintile 1 (most deprived)	151 (8.7%)
	Quintile 2	432 (24.8%)
	Quintile 3	326 (18.7%)
	Quintile 4	447 (25.7%)
	Quintile 5 (least deprived)	382 (21.9%)
**Education status**	Group1 (No formal qualifications)	953 (54.7%)
	Group 2 (GCSE, Alevel, NVQ 1-3)	469 (26.9%)
	Group 3 (1st or higher degree, NVQ 4-5)	317 (18.2%)
**eGFR at study entry**	Mean (SD)	52.5 (10.4)
	> 60	418 (24.0%)
	45-59	911 (52.3%)
	30-44	386 (22.2%)
	<30	26 (1.5%)
**Albuminuria**	No albuminuria	1456 (83.6%)
	Microalbuminuria (≥2.5 mg/mmol M, ≥3.5 mg/mmol F but <30 mg/mmol)	239 (13.7%)
	Macroalbuminuria (≥30 mg/mmol)	41( 2.4%)
**Diabetes**	Yes	294 (16.9%)
	No	1447 (83.1%)
**Hypertension**	On antihypertensive medication	1426 (81.9%)
	BP > 140/90 at study assessment, but not on antihypertensive medication	102 (5.9%)
	No hypertension	213 (12.2%)
**Number of antihypertensive medications**	None	315 (18.1%)
	1	615 (35.3%)
	2	488 (28.0%)
	3 or more	323 (18.6%)
**Taking RAASi**	Yes	1123 (64.5%)
	No	618 (35.5%)
**History of CVD**	Yes	592 (34.0%)
	No	1149 (66.0%)
**Smoking**	Current	81 (4.7%)
	Ex-smoker	866 (49.7%)
	Never	794 (45.6%)
**Alcohol**	No alcohol	711 (40.8%)
	Drinking within recommended limits	877 (50.4%)
	Drinking above recommended limits	65 (3.7%)
**BMI**	Normal or underweight	353 (20.3%)
	Overweight	738 (42.4%)
	Obese	650 (37.3%)
**Central obesity**	Yes	1480 (85.0%)
	No	260 (14.9%)
**Total chol:HDL ratio**	>4.5	306 (17.6%)
**Aware of CKD diagnosis**	Yes	1026 (58.9%)
	No	715 (41.1%)

Abbreviations:

CKD chronic kidney disease.

BP blood pressure.

eGFR estimated Glomerular Filtration Rate.

GCSE General certificate of Secondary Education.

A level Advanced level.

NVQ National Vocational Qualification.

RAASi renin-angiotensin aldosterone system inhibitors.

CVD cardiovascular disease.

### Antihypertensive treatment

**Figure 1:**
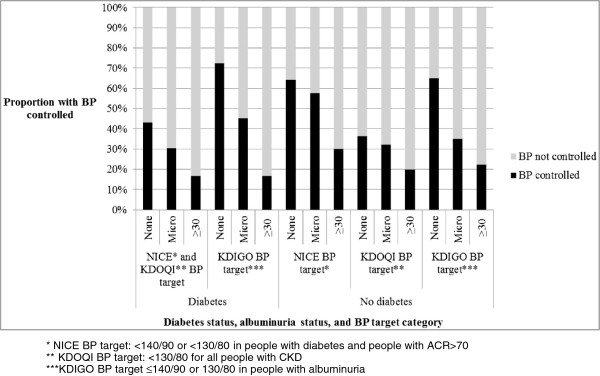
BP control by diabetes and albuminuria status in 1426 people with CKD 3 and hypertension.

In those taking antihypertensive medication, renin-angiotensin aldosterone system inhibitors (RAASi) were the most commonly used (78.8% of patients). Of those on antihypertensives, 85/98 (86.7%) people who met the NICE CKD criteria for requiring RAASi (diabetes with any albuminuria, no diabetes with macroalbuminuria) were taking them. Among people taking only one agent (n = 615), 425 (69.1%) were taking RAASi, 62 (10.1%) were taking calcium channel blockers, 61 (9.9%) were taking beta blockers, and 59 (9.6%) were taking thiazide diuretics. Mean (±SD) BP for people on antihypertensive agents was 134 (±18) / 72 (±11) mmHg. The NICE BP control target was achieved in 829/1426 (58.1%), the KDOQI target in 512/1426 (35.9%), and the KDIGO target in 859/1426 (60.2%) (Table 2). BP control varied by diabetes status with 106/276 (38.4%) of people with diabetes achieving the NICE or KDOQI target (targets are the same in diabetes). 723/1150 (62.9%), 407/1150 (35.3%) and 695/1150 (60.4%) of people without diabetes achieved the NICE, KDOQI, and KDIGO targets respectively (Table 2 and Figure 1). In both people with diabetes and without, optimal control was less likely in those with albuminuria (Chi-squared test for trend in non-diabetics = 7.68, p = 0.006, and in diabetics = 8.59, p = 0.003) (Figure 1).

**Table 2:** Blood pressure control by albuminuria and diabetes status among people on antihypertensive medication

		**Diabetes, n = 276**				**No diabetes, n = 1150**				**Total n (%)**
		**n (column%) unless otherwise stated**				**n (column%) unless otherwise stated**				
**Albuminuria status**		**None (n = 195)**	**Micro albuminuria (n = 63)**	**Macro albuminuria (n = 18)**	**Subtotal diabetes**	**None (n = 981)**	**Micro albuminuria (n = 149)**	**Macro albuminuria (n = 20)**	**Subtotal no diabetes**	**n = 1426**
**Mean BP (SD)**	Systolic	132 (17)	140 (20)	155 (22)	135 (19)	133 (18)	136 (19)	141 (18)	135 (18)	134 (18)
	Diastolic	68 (10)	69 (10)	72 (11)	68 (10)	73 (11)	75 (11)	76 (9)	73 (11)	72 (11)
BP controlled (NICE target)*	Yes	84 (43.1)	19 (30.2)	3 (16.7)	106 (38.4)	631 (64.3)	86 (57.7)	6 (30.0)	723 (62.9)	829 (58.1)
	No	111 (56.9)	44 (69.8)	15 (83.3)	170 (61.8)	350 (35.7)	63 (42.3)	14 (70.0)	425 (37.0)	597 (41.9)
BP controlled (KDOQI target)**	Yes	84 (43.1)	19 (30.2)	3 (16.7)	106 (38.4)	355 (36.2)	48 (32.2)	4 (20.0)	407 (35.3)	512 (35.9)
	No	111 (56.9)	44 (69.8)	15 (83.3)	170 (61.8)	626 (63.8)	101 (67.8)	16 (80.0)	115 (68.9)	914 (64.1)
BP controlled (KDIGO target)***	Yes	141 (72.3)	21 (33.3)	3 (16.7)	165 (59.8)	639 (65.1)	52 (34.9)	4 (22.2)	695 (60.4)	859 (60.2)
	No	54 (27.7)	42 (40.4)	15 (83.3)	111 (40.2)	342 (34.9)	97 (65.1)	14 (77.8)	453 (39.4)	576 (39.8)
**Number of anti-hypertensive agents**	1	79 (40.5)	25 (39.7)	3 (16.7)	107 (38.9)	433 (44.1)	65 (43.6)	8 (40.0)	506 (44.1)	615 (43.1)
	2	54 (27.7)	19 (30.2)	5 (27.8)	78 (28.3)	345 (35.2)	61 (40.9)	4 (20.0)	410 (35.7)	488 (34.2)
	3 or more	62 (31.8)	19 (30.2)	10 (55.6)	90 (32.6)	203 (20.7)	23 (15.4)	8 (40.0)	232 (20.2)	323 (22.7)
**Taking RAASi**	Yes	173 (88.7)	56 (88.9)	15 (83.3)	243 (88.4)	750 (76.5)	112 (75.2)	17 (85.0)	877 (76.4)	1123 (78.8)
	No	22 (11.3)	7 (11.1)	3 (16.7)	32 (11.6)	231 (23.5)	37 (24.8)	3 (15.0)	271 (23.6)	303 (21.2)

* <140/90 or <130/80 in people with diabetes and people with ACR > 70.

** <130/80 for all people with CKD.

*** ≤ 140/90 or 130/80 in people with albuminuria.

Microalbuminuria defined as ACR > =2.5 mg/mmol (men), > = 3.5 mg/mmol (women) in at least two of the three urine specimens.

Macroalbuminuria defined as ACR ≥ 30 mg/mmol.

Abbreviations:

BP blood pressure.

RAASi renin-angiotensin aldosterone system inhibitors.

NICE National Institute for Health and Clinical Excellence.

KDOQI National Kidney Foundation Kidney Disease Outcome Quality Initiative.

ACR albumin/creatinine ratio.

### Factors associated with suboptimal BP control

**Figure 2:**
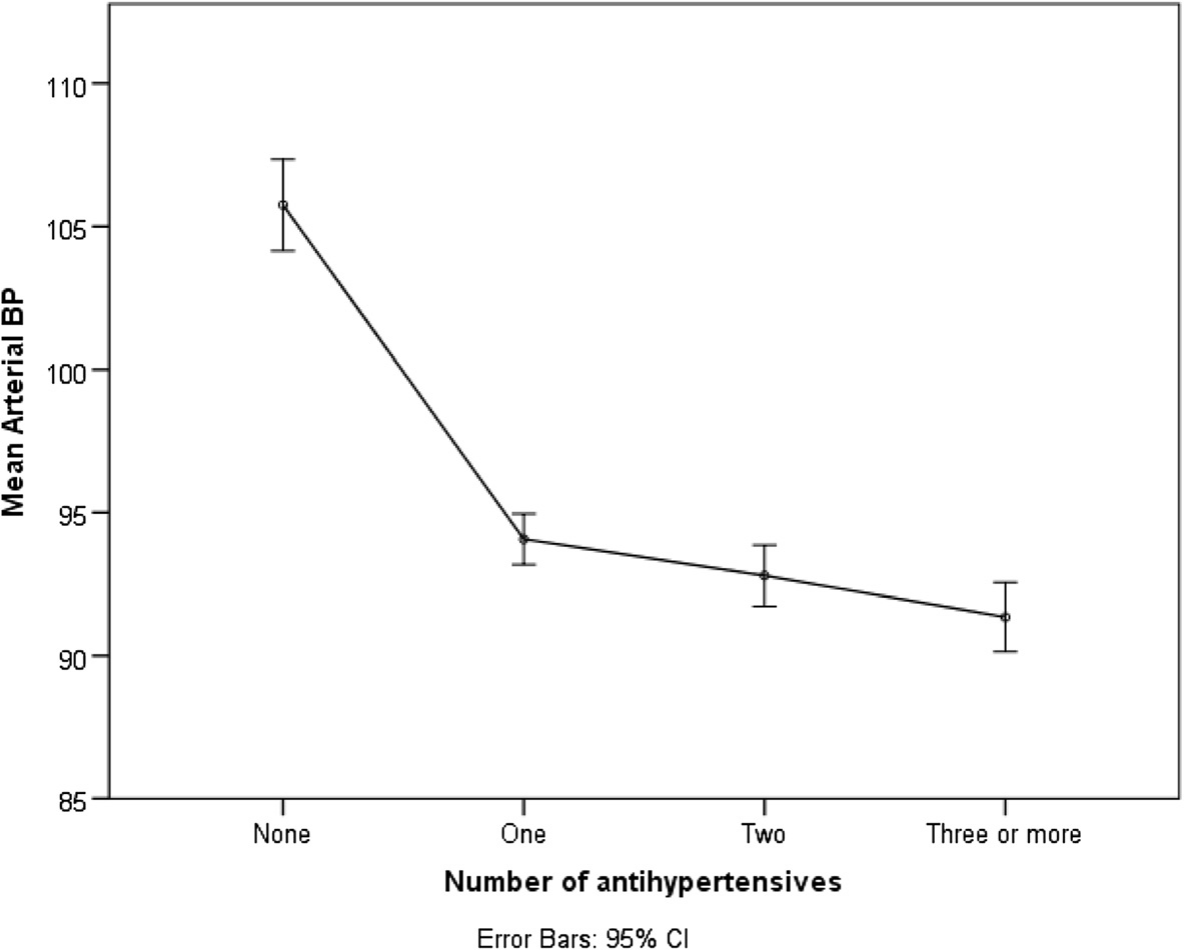
Number of antihypertensive medications and mean arterial BP in people with CKD 3 and hypertension.

On multivariable logistic regression analysis, older patients, those with diabetes, and those with albuminuria were less likely to achieve NICE BP targets whereas those with a history of cardiovascular disease were more likely to achieve them (Table 3). No difference was observed in these outcomes when CVD was excluded from the regression model. Findings for age and albuminuria were similar for KDOQI and KDIGO targets. The association with diabetes was not seen with either, but there was an association between lack of achievement of KDOQI target and male gender. All associations did not vary on sensitivity analysis in the population with eGFR < 60 at baseline, with the exception of the loss of the gender association with KDOQI targets. No association was seen with socioeconomic status, ethnicity, awareness of CKD diagnosis, alcohol intake, BMI, central obesity, or taking NSAIDs (data not shown). There was also no association between number of agents and achievement of BP control by any of the targets (NICE OR = 1.12 (95% CI 0.88,1.43), KDOQI OR 1.02 (95% CI 0.80, 1.31), and KDIGO OR 1.05 (95% CI 0.80,1.39)). Multivariable linear regression controlling for age, gender, albuminuria, previous CVD, and diabetes identified an association between number of antihypertensive drugs taken and lower MAP. For unit increase in number of antihypertensives, MAP dropped by 2.6 mmHg (95% CI 1.9,3.2, p < 0.01) (Figure 2). This effect was consistent when people with previous CVD were excluded from the analysis.

**Table 3:** Factors associated with achievement of BP targets in people on antihypertensive medication

		**Univariate odds ratios of achieving NICE BP targets**		**Multivariable odds ratios of achieving NICE BP target ∞**		**Univariate odds ratios of achieving KDOQI BP targets**		**Multivariable odds ratios of achieving KDOQI BP target †**		**Univariate odds ratios of achieving KDIGO BP targets**		**Multivariable odds ratios of achieving KDIGO BP target ∞**	
		**OR (95% CI)**	**p**	**OR (95% CI)**	**p**	**OR (95% CI)**	**p**	**OR (95% CI)**	**p**	**OR (95% CI)**	**p**	**OR (95% CI)**	**p**
**Sex (compared to female)**	Male	0.91 (0.73,1.12)	0.359	0.88 (0.71,1.10)	0.260	0.80 (0.64,1.00)	0.050	0.76 (0.60,0.96)	0.023	0.75 (0.61,0.93)	0.009	0.93 (0.74,1.18)	0.56
**Age (compared to <60 age group)**	60-69	0.60 (0.36,1.03)	<0.001	0.43 (0.26,0.71)	<0.001	0.73 (0.46, 1.18)	0.155	0.66 (0.41,1.08)	0.002	0.66 (0.39,1.12)	0.001	0.51 (0.29,0.89)	<0.001
	70-79	0.43 (0.26,0.71)		0.27 (0.17,0.43)		0.64 (0.41,1.01)		0.49 (0.31,0.79)		0.52 (0.32,0.87)		0.34 (0.20,0.58)	
	80+	0.35 (0.21,0.59)		0.21 (0.13,0.35)		0.60 (0.38, 0.97)		0.43 (0.26,0.72)		0.41 (0.24,0.68)		0.27 (0.15,0.48)	
**Diabetes (compared to people without diabetes)**	People with diabetes	0.36 (0.28,0.47)	<0.001	0.32 (0.25,0.43)	<0.001	1.12 (0.86,1.17)	0.410	1.08 (0.81,1.43)	0.601	0.97 (0.74,1.26)	0.796	1.14 (0.85,1.53)	0.38
**Albuminuria (compared to no albuminuria)**	People with albuminuria	0.54 (0.41,0.72)	<0.001	0.56 (0.42,0.74)	0.001	0.70 (0.52, 0.95)	0.021	0.65 (0.47,0.90)	0.009	0.70 (0.52,0.95)	0.021	0.21 (0.16,0.30)	<0.001
**History of CVD (compared to people with no CVD)**	People with CVD	1.41 (1.13,1.76)	0.002	1.87 (1.49,2.35)	<0.001	1.66 (1.33,2.07)	<0.001	1.89 (1.49,2.39)	<0.001	1.42 (1.14,1.77)	0.002	1.83 (1.43,2.34)	<0.001
**eGFR (compared to 45–59)**	60+	1.03 (0.81,1.31)	0.001	0.84 (0.65, 1.09)	0.088	0.78 (0.62,1.00)	0.145	-	-	0.97 (0.74,1.28)	0.004	0.83 (0.62,1.11)	0.27
	<45	0.66 (0.52, 0.83)		0.77 (0.60, 0.99)		0.92 (0.72, 1.17)				0.66 (0.52,0.85)		0.83 (0.64, 1.09)	

∞ Model adjusted for age, sex, albuminuria, diabetes, CVD, and eGFR.

† Model adjusted for age, sex, albuminuria, diabetes, and CVD.

Abbreviations:

BP blood pressure.

RAASi renin-angiotensin aldosterone system inhibitors.

NICE National Institute for Health and Clinical Excellence.

KDOQI National Kidney Foundation Kidney Disease Outcome Quality Initiative.

CVD cardiovascular disease.

eGFR estimated Glomerular Filtration Rate.

### Systolic and diastolic hypertension

Of the 597 people not controlled below the NICE target, 435 (72.9%) had isolated systolic hypertension (≥140), 13 (2.2%) had isolated diastolic hypertension (≥90), and 74 (12.4%) had both systolic and diastolic hypertension. Table 4 shows the distribution of systolic and diastolic blood pressure by age and eGFR in the whole study population. Table 5 shows the variation in systolic and diastolic hypertension (despite treatment) by age, and demonstrates the predominance of systolic hypertension in older age groups. Logistic regression analysis of the associations of achieving NICE systolic and diastolic targets separately demonstrated that older people had a lower odds ratio of achieving systolic targets (OR 0.17 (95% CI 0.09,0.32) p < 0.001 for over 80), and greater odds ratio of achieving diastolic targets (OR 2.35 (95% CI 1.11,4.96) p < 0.001 for over 80) compared to those under 60 years.

**Table 4:** Variation in systolic and diastolic blood pressure by age and eGFR in whole study population

			**Age**							
			**<60**		**60-69**		**70-79**		**80+**	
			**SBP**	**DBP**	**SBP**	**DBP**	**SBP**	**DBP**	**SBP**	**DBP**
			**mean (SD)**	**mean (SD)**	**mean (SD)**	**mean (SD)**	**mean (SD)**	**mean (SD)**	**mean (SD)**	**mean (SD)**
			**mmHg**	**mmHg**	**mmHg**	**mmHg**	**mmHg**	**mmHg**	**mmHg**	**mmHg**
eGFR at baseline	>60	n	40		148		174		56	
		BP	122.1 (15.0)	78.7 (9.8)	131.8 (15.6)	77.3 (10.0)	136.2 (16.6)	73.9 (9.8)	139.0 (19.3)	75.1 (10.9)
	45-59.99	n	68		231		412		200	
		BP	124.2 (15.6)	77.6 (10.1)	130.7 (16.4)	74.7 (11.1)	134.3 (16.6)	71.8 (10.6)	138.4 (21.4)	70 (11.1)
	30-44.99	n	19		63		164		140	
		BP	125.5 (22.1)	79.3 (12.73)	133.8 (17.4)	74.7 (11.8)	135.0 (21.0)	69.0 (10.7)	137.5 (20.7)	69.2 (11.1)
	<30	n	1		3		11		11	
		BP	111.3	73.7	137.6 (13.4)	68 (16.1)	134.1 (14.4)	72.5 (9.0)	136.1 (19.1)	65.0 (5.49)

**Table 5:** Variation in systolic and diastolic hypertension by age

		**Proportion with isolated systolic hypertension (SBP >140)**	**Proportion with isolated diastolic hypertension (DBP >90)**	**Proportion with both systolic and diastolic hypertension (SBP >140 and DBP > 90)**	**Proportion with normal blood pressure (both SBP < 140 and DBP <90)**	**Total**
Age group	<60	7.8%	4.7%	7.0%	80.5%	100%
	60-69	20.7%	1.8%	8.1%	69.4%	100%
	70-79	32.9%	0.7%	3.7%	62.8%	100%
	80+	39.8%	0.2%	4.2%	55.8%	100%

SBP = systolic blood pressure.

DBP = diastolic blood pressure.

## Discussion

In this cohort of 1741 people with CKD stage 3, hypertension was common with a prevalence of 88% and BP control was suboptimal, with 42% not achieving the NICE BP target, 40% not achieving the KDIGO BP target, and 64% not achieving the more strict KDOQI BP target. Presence of diabetes and higher levels of albuminuria were associated with a smaller proportion of people achieving BP control targets. After adjustment for potential confounding factors, poor BP control was associated with increasing age and albuminuria for all three BP target groups and with diabetes in the NICE BP target group. Older age was associated with better diastolic control and poorer systolic control. Better BP control was associated with past history of CVD. The majority of patients were on one or two antihypertensive medications (most commonly RAASi) and taking a greater number of antihypertensive medications was associated with lower MAP.

The prevalence of hypertension in this cohort is similar to other studies. In the Chronic Renal Insufficiency Cohort (CRIC) Study, it was between 82% and 91% in people with eGFR between 30 and 59 mL/min/1.73 m^2^[33]. In the Kidney Early Evaluation Programme (KEEP) and the National Health and Nutrition Examination Survey (NHANES), the prevalence of hypertension was between 84 and 92% for people with eGFR between 40 and 60 mL/min/1.73 m^2^[9,11]. As with previous studies, systolic hypertension predominated in the uncontrolled group with hypertension [12]. The findings of poor BP control in older people, people with diabetes, and people with albuminuria are consistent with previous studies [9,31,32]. For the KDOQI target, we identified similar association with male gender identified in the Kidney Early Evaluation Program (KEEP) cohort, but not the association with obesity identified in KEEP [9,15]. The predominantly white population in this study limited our ability to draw conclusions about the association between CKD-related hypertension and ethnicity identified in other studies [9,15,31,34]. The main concern is that people at greater cardiovascular risk (older people and people with diabetes and/or albuminuria) and people at greater risk of progression (with diabetes and albuminuria) were less likely to achieve BP targets. In the UK in 2010/11, the mean practice-level achievement of the QOF target for blood pressure control in CKD patients was 74.9% (standard deviation (SD) 8.2%), and the median 74.7% [23]. Our study has shown that BP control may be considerably worse than that when individual patient data are analysed and more robust targets are adopted [19,21].

Recent data from the National Diabetes Audit in England showed that only 36.4% of people with diabetes were achieving target blood pressure (<140/80 if no co-morbidity, <130/80 with comorbidity, including CKD) [35]. Our study findings are very similar (38.0% of people with diabetes achieving BP targets) and add information on BP control by albuminuria status and in people without diabetes (Table 2).

There is evidence that RAAs inhibitors (RAASi) reduce progression of CKD in patients with diabetic nephropathy and in those with non-diabetic CKD and macroalbuminuria, [36,37] although a recent Cochrane review has not been able to identify sufficient evidence to determine the effectiveness of RAASi in patients with stage 1 to 3 CKD who do not have diabetes [38]. In the UK, NICE guidelines recommend offering RAASi to non-diabetic people with CKD and hypertension if they have macroalbuminuria (ACR > =30 mg/mmol), or to people with diabetic nephropathy who have ACR >2.5 mg/mmol in men or 3/5/mmol in women [19]. RAASi were being taken by the majority of relevant participants in this study.

Despite not finding a significant association between numbers of hypertensive agents and achievement of optimal BP control, this study demonstrated that a large proportion of people were only taking one agent. Furthermore, there was an independent association between the number of antihypertensives and lower MAP, even when previous CVD was excluded (to remove indication bias). This suggests there is scope for improving BP control by the use of more antihypertensive agents in combination. The lack of association of agent number and optimal control may be as a result of indication bias, as many older patients have multiple co-morbidities. In recommendations to add more agents, risk of side effects, impact on quality of life, and costs all need to be considered, as well as issues of medication adherence. The potential for RAASi to precipitate acute kidney injury, for example, is an important factor requiring evaluation in this context. BP control and these related concerns will be important aspects for the proposed UK national CKD audit in primary care.

Optimal targets for BP control remain the subject of debate. Furthermore the correct management of isolated systolic hypertension is uncertain and very low blood pressure has been associated with poor outcomes, particularly in diabetes [39]. Further research would be valuable in the context of CKD. In this analysis we applied the two most widely applied evidence-based guidelines in use at the time that the study was conducted, NICE and KDOQI, and added analysis for the KDIGO guidelines in view of their current relevance.

This study had several strengths, including large numbers of people with CKD, being conducted in a primary care setting, standardisation of blood pressure and other measures, and the use of three morning urine samples to assess albuminuria. However, it also has several limitations, including its cross sectional design, which limits the ability to infer causality (although longitudinal follow up of this cohort will allow assessment of the effect of baseline BP on outcome, and of change in BP management on control). It is possible that people taking a single antihypertensive agent were taking it for other reasons, and that the observed relationship between CVD and improved BP control could be a reflection of reverse causality. We checked this among people with heart failure taking only one agent and identified only 25 people whose blood pressure was <140/90. We conclude that the risk of bias from people on single agents for reasons other than hypertension was therefore low. A further potential limitation is that a significant proportion of the study population (24%) were found to have an eGFR ≥60 at baseline, which might be considered to question their CKD diagnosis. However, all the participants met the formal definition for CKD prior to inclusion (including chronicity of low eGFR) and, importantly, were therefore on CKD registers in their respective GP practices. We therefore included them in the analyses to improve the generalisability of these findings to normal practice circumstances. There is also potential that non response to recruitment could have caused selection bias, and that the predominantly elderly population could result in survivor bias. The potential for selection bias means that caution should be used in application of these results to general populations with CKD. In addition, we cannot comment on whether people on antihypertensive treatment were receiving adequate doses of medication or adhering. Optimisation of drug dosage might therefore represent a potential area of improvement not assessed in this study. This study under-represents ethnic minorities and the findings should therefore be interpreted with caution in different ethnic groups. In addition, the study population is not representative of the general population due to the high proportion of older people. However, it included a range of general practices from urban and rural locations, and hence is broadly representative of people with CKD stage 3 in primary care in the UK. It therefore highlights the challenges of BP control, and the importance of measuring urine ACR and using it to guide intervention in patients with moderate CKD.

## Conclusions

Failure to achieve BP targets was common in CKD patients with hypertension in this study, particularly in those at highest risk, and systolic hypertension predominated in those with uncontrolled BP. These findings suggest that there is scope for improving BP control in CKD stage 3 in primary care, possibly using more antihypertensive agents in combination, though there is a need to weigh potential side effects and costs.

## Competing interests

The authors declare that they have no financial or non financial competing interests.

## Authors’ contributions

SF designed and conducted the analyses and was the primary author. PR and MT helped to design the analyses and provided critical manuscript review. NM carried out the data collection and provided critical manuscript review. SH advised on statistical analyses. NM, CM, MT, and RF designed the RRID study and provided critical manuscript review. All authors read and approved the final manuscript.

## Pre-publication history

The pre-publication history for this paper can be accessed here:

http://www.biomedcentral.com/1471-2296/14/88/prepub

## Supplementary Material

Additional file 1: Table S1 Characteristics of people with and without hypertension. Click here for file
