# Supplementary material for: Suboptimal blood pressure control in chronic kidney disease stage 3: baseline data from a cohort study in primary care
Source: BMC Fam Pract. 2013 Jun 24;14:88. doi: 10.1186/1471-2296-14-88 (PMC3701497; doi:10.1186/1471-2296-14-88)
Supplement: Additional file 1: Table S1 — Characteristics of people with and without hypertension. [file 1471-2296-14-88-S1.docx]

**Supplementary Table. Characteristics of people with and without pre-existing hypertension or identified as hypertensive at baseline in the RRID study**

|  |  | **Hypertension or identified at baseline**  **n=1528** |  | **No hypertension**  **n=213** |  | **Total**  **n=1741** |
| --- | --- | --- | --- | --- | --- | --- |
|  | **Categories** | **Number** | **%** | **Number** | **%** |  |
| **Gender** | Male | 634 | 92% | 55 | 8% | 689 |
|  | Female | 894 | 85% | 158 | 15% | 1052 |
| **Age** | <60 | 92 | 72% | 36 | 28% | 128 |
|  | 60-69 | 372 | 84% | 73 | 16% | 445 |
|  | 70-79 | 689 | 91% | 72 | 9% | 761 |
|  | 80+ | 375 | 92% | 32 | 8% | 407 |
| **Ethnicity** | White | 1488 | 88% | 210 | 12% | 1698 |
|  | Other | 40 | 93% | 3 | 7% | 43 |
| **Aware of CKD diagnosis at baseline** | Yes | 904 | 88% | 122 | 12% | 1026 |
|  | No | 624 | 87% | 91 | 13% | 715 |
| **Deprivation (IMD quintile)** | 1 (most deprived) | 130 | 86% | 21 | 14% | 151 |
|  | 2 | 370 | 86% | 62 | 14% | 432 |
|  | 3 | 294 | 90% | 32 | 10% | 326 |
|  | 4 | 397 | 89% | 50 | 11% | 447 |
|  | 5 (least deprived) | 334 | 87% | 48 | 13% | 382 |
| **Education status** | Group 1 (no formal education) | 836 | 88% | 117 | 12% | 953 |
|  | Group 2 | 411 | 88% | 58 | 12% | 469 |
|  | Group 3 (highest education status) | 279 | 88% | 38 | 12% | 317 |
| **PMH CVD** | Yes | 547 | 92% | 45 | 8% | 592 |
|  | No | 981 | 85% | 168 | 15% | 1149 |
| **Diabetes** | Yes | 286 | 97% | 8 | 3% | 294 |
|  | No | 1242 | 86% | 205 | 14% | 1447 |
| **Smoking** | Current smoker | 64 | 79% | 17 | 21% | 81 |
|  | Ex smoker | 767 | 89% | 99 | 11% | 866 |
|  | Never smoker | 697 | 88% | 97 | 12% | 794 |
| **Alcohol** | None | 625 | 88% | 86 | 12% | 711 |
|  | Within recommended limits | 772 | 88% | 105 | 12% | 877 |
|  | Above recommended limits | 61 | 94% | 4 | 6% | 65 |
| **BMI** | Underweight (<18.5) | 3 | 60% | 2 | 40% | 5 |
|  | Normal (18.5-25) | 284 | 82% | 64 | 18% | 348 |
|  | Overweight (25-30) | 642 | 87% | 96 | 13% | 738 |
|  | Obese (>30) | 599 | 92% | 51 | 8% | 650 |
| **Central obesity** (by IDF race-specific) categories of waist circumference | Centrally obese | 1316 | 89% | 164 | 11% | 1480 |
|  | Not centrally obese | 211 | 81% | 49 | 19% | 260 |
| **Albuminuria** | At least microalbuminuria | 267 | 95% | 13 | 5% | 280 |
|  | No albuminuria | 1257 | 86% | 199 | 14% | 1456 |
| **eGFR** | >60 | 336 | 80% | 82 | 20% | 418 |
|  | 45-59 | 799 | 88% | 112 | 12% | 911 |
|  | <45 | 393 | 95% | 19 | 5% | 412 |
